# Falling Third Trimester Insulin Requirements and Adverse Pregnancy Outcomes in Individuals with Pre-Existing Diabetes: A Retrospective Cohort Study

**DOI:** 10.3390/jcm14217737

**Published:** 2025-10-31

**Authors:** Marina Vainder, Navneet Natt, Parastoo Sayyar, Ambreen Syeda, Rizwana Ashraf, Nicholas Mitsakakis, Denice S. Feig, John Kingdom, Rohan D’Souza

**Affiliations:** 1 Department of Obstetrics and Gynaecology, Mount Sinai Hospital, University of Toronto, Toronto, ON M5G 1X5, Canada; 2 Department of Obstetrics and Gynaecology, McMaster University, 1280 Main Street West, Hamilton, ON L8S 4L8, Canada; 3 Department of Health Research Methods Evidence and Impact, McMaster University, Hamilton, ON L8S 4L8, Canada; 4 Clinical Research Unit, Children’s Hospital of Eastern Ontario Research Institute, Ottawa, ON K1H 8L1, Canada; 5 Department of Medicine, Division of Endocrinology, University of Toronto, Toronto, ON M5S 3H2, Canada; 6 Institute of Health Policy, Management and Evaluation, University of Toronto, Toronto, ON M5T 3M6, Canada

**Keywords:** type 1 diabetes, type 2 diabetes, diabetes in pregnancy, insulin requirement

## Abstract

**Objective:** To determine whether a third-trimester drop in insulin requirements in pregnant people with pre-existing diabetes is associated with a subsequent occurrence of adverse pregnancy outcomes. **Research Design and Methods:** We conducted a retrospective cohort study of patients with type 1 and 2 diabetes who were followed at a tertiary referral center in Toronto, Canada. We collected data on insulin dosing in the third trimester (after 28 weeks of pregnancy) and compared outcomes in those with and without a third-trimester drop of 15% or more in their total insulin requirements. Our primary outcome was a composite of stillbirth, spontaneous preterm birth or preterm premature rupture of membranes, and iatrogenic preterm birth or cesarean birth for fetal wellbeing concerns, occurring following the drop in insulin requirements. We conducted regression analyses controlling for early pregnancy glycosylated hemoglobin, body mass index, and diabetes-related microvascular disease, and presented results as odds ratios (OR) with 95% confidence intervals (95%CI). **Results:** We included 350 pregnant people—146 with type 1 and 204 with type 2 diabetes. Of these, 54 (15.4%) had a third-trimester drop of 15% or more in their total insulin requirements. There was no difference in the primary outcome between groups (OR 0.97; 95% CI 0.41–2.10). **Conclusions:** Based on this single-center study, limited by sample size and analytic constraints, in people with pre-existing diabetes, a third-trimester drop of ≥15% in total insulin requirements was not associated with subsequent occurrence of adverse pregnancy outcomes. Larger prospective studies looking at associations between a drop in insulin requirements and subsequent occurrence of adverse pregnancy outcomes are necessary to inform meta-analyses and clinical decision making.

## 1. Introduction

Pre-existing diabetes (type 1 and type 2) accounts for 13–20% of all diabetic pregnancies and is on the rise [1,2]. These pregnancies are associated with adverse perinatal outcomes including preeclampsia, intrauterine fetal growth restriction, preterm birth, stillbirth, and neonatal morbidity [3,4]. Management of diabetes in pregnancy is challenging due to limited treatment options, with insulin remaining the mainstay of therapy throughout pregnancy [5,6,7]. As pregnancy progresses especially during the second and third trimesters, insulin requirements (IR) have been observed to increase [8,9,10,11,12]. This is attributed to a rise in placental synthesis of diabetogenic hormones, including estrogen, progesterone, human placental lactogen (hPL), and cortisol [13,14,15], and is believed to be adaptive as it allows for delivery of sufficient glucose to the growing fetus [16]. However, in 8–25% of the pregnancies IR actually declines in late pregnancy [8,17,18,19,20].

Clinically, there is a concern that this decrease in IR is attributable to a decline in the synthesis of placental hormones hPL and progesterone, and therefore represents placental insufficiency [21]. Since these hormones play a key role in mediating insulin resistance during pregnancy, an unexpected decline in insulin requirements during the third trimester may be indicative of reduced placental function [13]. Although a decline in insulin requirements during the third trimester has well-established pathophysiological relevance in gestational diabetes mellitus (GDM) where it is often considered a failing fetoplacental unit due to the typically insulin resistant state of late pregnancy [22], it has also been observed in pregnant people with type 1 and type 2 diabetes. Several retrospective studies have reported associations between declining insulin needs and adverse outcomes in women with pre-existing diabetes [17,18,19,20].

However, while some studies have drawn an association between a decrease in IR and adverse pregnancy outcomes related to placental dysfunction [19,20], others have been unable to show an association [18,21,23,24,25,26]. This discrepancy could be the result of variations in the choice and measurement of exposures and outcomes, the small numbers of included patients, the inclusion of pre-existing and gestational diabetes, and the failure to account for temporality in the associations between the exposure and outcomes [27]. Thus, there remains uncertainty regarding whether a drop in third-trimester IR that some pregnant people with pre-existing diabetes experience should warrant early delivery, increased maternal-fetal monitoring, or no additional intervention.

The aim of our study was to elucidate the relationship between a third trimester drop in IR and a subsequent occurrence of adverse pregnancy outcomes in patients with pre-existing diabetes.

## 2. Research Design and Methods

We conducted a retrospective cohort study, in which we included consecutive patients with type 1 or type 2 diabetes with singleton pregnancies that continued beyond the third trimester, i.e., 28 weeks of gestation, who were followed at the Diabetes in Pregnancy clinic at a tertiary care center in Toronto, Ontario, Canada between the years 2000 and 2016. Only patients who were taking insulin during pregnancy were included. Eligible patients were identified through the Diabetes in Pregnancy clinic database. Data on serial insulin requirements, as well as pregnancy, labor, and delivery outcomes were obtained from electronic medical records and paper charts.

We excluded pregnant people with gestational diabetes from our analysis given the pathophysiology of this condition is different from that of pre-gestational diabetes, pregnancies with a diagnosis of major fetal anomalies and those for whom third-trimester insulin dosing or pregnancy outcomes were unavailable. The study was approved by the institutional Research Ethics Board (REB #17-0057-C).

For each eligible participant, we collected data on demographics, reproductive and past medical history, hemoglobin A1C at the start of pregnancy, medical complications of diabetes, insulin dosing at each visit, course of the index pregnancy, fetal surveillance, birth data, and short-term neonatal outcomes. We have also gathered data on serial insulin requirements at the first clinic visit in the first trimester of pregnancy and every subsequent visit in the third trimester of pregnancy from 28 weeks of gestation onwards. Patients were usually followed up after every 2 to 4 weeks in the diabetic clinic. At each visit, we collected data on daily IR, which included total, basal, and bolus insulin units. For patients who were on an insulin pump, we gathered data from the pump and calculated average daily basal and bolus insulin requirements in units. We excluded pregnancies where data were presented as the carbohydrate-to-insulin ratio and the total intake of carbohydrate during the particular week was missing, as we were not able to calculate the insulin value in units. Ultimately, we calculated the percentage drop in IR for each consecutive visit in the third trimester by calculating the percent fall in insulin dosage (PFID) from the peak total insulin dose (PTID), defined as the highest total insulin dose (TID) in pregnancy, and the trough total insulin dose (TTID), defined as the lowest TID following the peak, using the following formula: PFID = (PTID − TTID)/PTID × 100. We did not include the period within 5 days of antenatal steroid administration when calculating the PTID. All those who had a 15% or greater drop in their third trimester total IR were defined as our cases, while all other patients served as controls. We then repeated the analysis using a 30% or greater drop in third trimester total IR as the cut off between cases and controls.

Only outcomes occurring *subsequent to* the drop in IR that could be related to the drop in IR were considered. For example, we only considered a stillbirth or spontaneous preterm birth that occurred *after* the third-trimester drop in IR. Pre-existing conditions or conditions in evolution that were likely to be the cause rather than the effect of the falling IR such as preeclampsia and fetal growth restriction were not included as outcomes.

We chose the 15% and 30% drop in IR based on the published literature where four studies on this topic used a 15% threshold [18,19,20,25] and one study used a 30% threshold to define a significant drop in IR [24]. Similarly, we chose total insulin dose for our primary analysis since this was used in the majority of published studies [19,20,24,25,26]. Since only one study used a weight adjusted basal insulin dose [18], this formed our secondary analysis.

Based on a discussion with a multidisciplinary team of clinical experts, we used a composite primary outcome of acute adverse events that occurred *after* a third-trimester drop in IR using universally accepted definitions: (1) third-trimester stillbirth (fetal demise), (2) spontaneous preterm birth or preterm premature rupture of membranes (prior to 37 weeks of gestation), (3) iatrogenic preterm birth for fetal wellbeing concerns other than if labor was induced for the sole indication of falling IR, or (4) unplanned cesarean birth for fetal wellbeing concerns, but not merely because of falling IR. The secondary outcomes were hypertensive disorders of pregnancy, gestational age at birth, mean birthweight, birthweight below 10th centile, and neonatal intensive care unit admission.

We performed statistical analysis using R version 4.5.1. In particular, for epidemiological analyses, we used the epiR package (version 2.0.85). We calculated proportions of composite outcomes in the two cohorts and compared them using Fisher’s exact tests. A *p* value of <0.05 was considered significant. We performed multivariable logistic regression analysis, controlling for pre-pregnancy or early pregnancy hemoglobin A1c 7, maternal body mass index (BMI), and pre-existing microvascular diseases likely related to diabetes, which included nephropathy, neuropathy, and retinopathy. Odds ratios (OR) were reported with 95% confidence intervals (CIs). We handled missing data using a complete-case analytic approach.

## 3. Results

**Figure 1:**
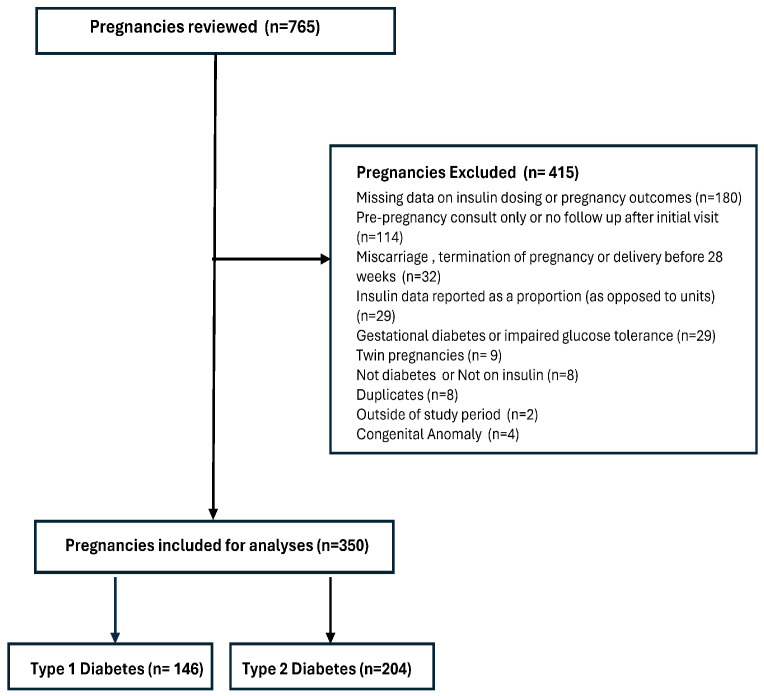
Study Flowchart.

We reviewed 765 pregnancies with pre-existing diabetes over a 16-year period. Of these, 415 pregnancies were excluded for various reasons including incomplete data on insulin dosage or pregnancy outcome, failure to follow-up after the initial visit, miscarriage or termination of pregnancy, delivery before 28 weeks of gestation, insulin data reported as a proportion (rather than absolute units), or a diagnosis of gestational diabetes or impaired glucose tolerance. Full details of exclusions are shown in Figure 1.

**Figure 2:**
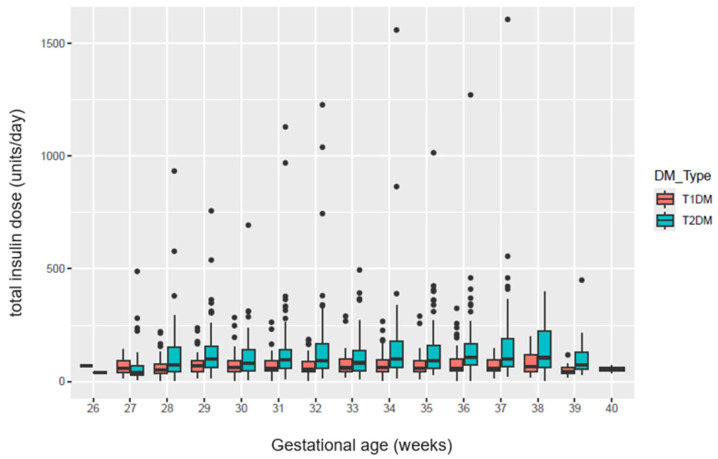
Distribution of total daily insulin dose (units) during third trimester in pregnant people with type 1 and type 2 diabetes.

The final cohort included 350 singleton pregnancies with pre-existing diabetes, of which 146 (41.7%) had type 1 diabetes and 204 (58.3%) had type 2 diabetes. Of the pregnancies included, 54 (15.34%) had a drop of ≥15% in their third trimester total insulin requirements, and 22 (6.3%) participants had a drop of ≥30%. Figure 2 presents the total insulin dose required in units/day during the third trimester in type 1 and type 2 diabetic pregnancies.

A comparison of maternal baseline and pregnancy characteristics between groups with and without a drop in total IR ≥ 15% is summarized in Table 1. Pregnant people with a ≥15% drop in total IR were more likely to have type 1 diabetes (66.6% vs. 37.1%, *p* < 0.001), lower BMI (26.7 ± 7.0 vs. 29.7 ± 7.3, *p* = 0.005), and were less likely to be nulliparous (35.2% vs. 55.4%, *p* = 0.010). No significant differences were observed in terms of maternal age, smoking status, pre-pregnancy HbA1c, pre-existing microvascular disease related to diabetes, or pre-existing hypertension.

When stratified by type of diabetes, in pregnant people with type 1 diabetes, those with a ≥15% insulin drop had a significantly lower BMI (24.3 vs. 26.9 kg/m^2^, *p* = 0.029) and were less likely to be nulliparous (25.0% vs. 49.1%, *p* = 0.019), with no other significant differences. Among pregnant people with type 2 diabetes, characteristics were similar between the two groups except for a higher rate of nephropathy in those with a ≥15% drop in total daily IR (11.1% vs. 1.1%, *p* = 0.041). No statistically significant differences were seen in age, BMI, HbA1c, or other microvascular diseases (Supplementary File S1 (Tables S1 and S2)).

In terms of pregnancy outcomes, no statistically significant differences were observed in composite or component pregnancy outcomes between people with ≥15% and <15% reductions in total daily IR during pregnancy. This finding was consistent across the overall cohort and when stratified by diabetes type (type 1 and type 2). Rates of stillbirth, preterm birth, hypertensive disorders, mean neonatal birthweight, and NICU admission were comparable between groups (*p* > 0.05). The proportion of infants with birthweight below the 10th percentile was also similar between groups, occurring in 5.6% of the case group and 8.1% of the control group (*p* = 0.707), indicating no statistically significant difference. Pregnancy outcomes stratified by an overall 15% drop in total IR are summarized in Table 2 while pregnancy outcomes stratified by type of diabetes are described in Supplementary File S2 (Tables S3 and S4).

The demographic characteristics and outcomes stratified by a 30% drop in total IR are summarized in Table 3 and Table 4, respectively. In summary, pregnant people with a ≥30% drop in total IR were also more likely to have type 1 diabetes (63.6% vs. 40.4%, *p* = 0.05). There were no significant differences in terms of maternal age, BMI, parity, smoking status, pre-pregnancy HbA1c, pre-existing microvascular disease related to diabetes, or pre-existing hypertension. In terms of pregnancy outcomes, no significant differences were observed in the composite or secondary outcomes between the two groups.

When stratified by type of diabetes, in pregnant people with type 1 diabetes, those with a ≥30% insulin drop had a significantly lower proportion of nulliparous people compared to controls (14.3% vs. 46.2%, *p* = 0.044), with no other significant differences.

Among pregnant people with type 2 diabetes, characteristics were similar between the two groups (Supplementary File S3 (Tables S5 and S6)). In terms of pregnancy outcomes, no statistically significant differences were observed in composite or component pregnancy outcomes between people with ≥30% and ≤30% reductions in total daily IR during pregnancy when stratified by diabetes type (Supplementary File S4 (Tables S7 and S8)).

Compared with controls, there was no significant difference in the frequency of the composite adverse outcome between those with a ≥15% drop in total IR (OR 0.97; 95% CI 0.41–2.10), or a ≥30% drop in total IR (OR 0.73; 95% CI 0.16–2.3).

When this analysis was restricted to pregnancies with type 1 diabetes, there was still no difference in the frequency of the composite adverse outcome between those with a ≥15% drop in total IR (OR 1.28; 95% CI 0.43–3.52), or a ≥30% drop in total IR (OR 1.45; 95% CI 0.29–5.54) Similarly, there was no significant difference in the frequency of the composite adverse outcome among those with type 2 diabetes with a ≥15% drop in total IR (OR 0.55; 95% CI 0.08–2.30). For ≥30% drop in IR, odds ratio could not be estimated.

For our secondary analysis, a comparison of maternal baseline and pregnancy characteristics between groups with and without a drop in basal IR ≥ 15% is summarized in Supplementary File S5 (Table S9). There were no differences between groups in terms of maternal age, BMI, parity, smoking status, pre-pregnancy HbA1c, pre-existing microvascular disease related to diabetes, or pre-existing hypertension. Pregnancy outcomes stratified by a 15% drop in basal IR are summarized in Supplementary File S5 (Table S10). The demographic characteristics and outcomes stratified by a 30% drop in basal IR are summarized in Supplementary File S6 (Tables S11 and S12).

Compared with controls, there was no difference in the frequency of the composite adverse outcome between those with a ≥15% drop in basal IR (OR 0.75; 95% CI 0.27–1.81), or a ≥30% drop in basal IR (OR 0.74; 95% CI 0.11–2.88).

Within our cohort, 7 (2%) patients were delivered based solely on a finding of what was deemed to be a clinically significant drop in insulin requirements. Out of these, one patient had a ≥15% drop in total IR. According to the study protocol, these cases were not included in our analysis. There were three antepartum stillbirths at 36 + 0, 38 + 4, and 38 + 5 weeks gestation, all three of which were in the control group.

Given the small number of events observed for secondary outcomes, a post hoc power calculation was performed. Assuming an expected event rate of the composite outcome is 20% in the <15% drop group and aiming to detect an absolute increase to 25% in the ≥15% drop group, the calculated power was approximately 12%.

## 4. Discussion

In this retrospective study of pregnant people with pre-existing diabetes, we found no differences in the composite adverse pregnancy outcome related to placental insufficiency between those who with and without a drop in their third trimester total or basal insulin requirements. This was true for both a drop of ≥15% and a drop of ≥30%.

Our study findings are in contrast to a previously published study in 2014 which reported a higher incidence of the composite outcome of markers of placental dysfunction, which included preeclampsia, SGA, stillbirth or placental abruption, and birth before 30 weeks following a third-trimester drop in total IR (defined as ≥15% from the highest insulin dose to the lowest), and when compared with those that did not experience a third-trimester drop in IR. This increase was driven by an increase in preeclampsia and SGA [20]. A subsequent prospective study by the same group showed a correlation between a drop in IR after 20 weeks gestation and a composite of clinical markers of placental dysfunction [19]. More specifically, a ≥15% drop in IR was associated with an increased risk of the composite primary outcome which included preeclampsia. The authors concluded that falling IR is an important clinical sign among women with preexisting diabetes that should alert the clinician to investigate underlying placental dysfunction [19].

There are several possible reasons for the differences in the results. First, our composite outcome was restricted to events occurring after the drop in IR and only included components that can be attributed to placental dysfunction and considered as markers of fetal distress which included stillbirth, spontaneous preterm birth, iatrogenic preterm birth, or cesarean section for fetal wellbeing concerns. We did not include fetal growth restriction and preeclampsia that are suggestive of placental insufficiency in our composite outcomes because these conditions are relatively “chronic” making it impossible to determine whether the falling IR was the cause or effect of these events. Second, compared to the earlier studies, our study included a slightly higher proportion of type 1 diabetes and our glycemic control targets of <5.3 mmol/L fasting, and <6.7 mmol/L at 2 h post prandial, were slightly stricter. It is possible that our tighter glycemic targets could have reduced adverse outcomes.

Our study findings align with some other studies that found no correlation between a drop in IR and adverse pregnancy outcomes [18,21,23,24,25,26]. A systematic review that includes all published studies including data from this publication, and which used the Grading of Recommendations, Assessment, Development, and Evaluation (GRADE) approach to determine the certainty of evidence for associations, demonstrated an important association between a third trimester drop in IR, and preeclampsia with high certainty of evidence and neonatal respiratory distress with moderate certainty of evidence. There was low to very low certainty of evidence for an important association between a drop in IR, preterm birth, and a composite of clinical outcomes reflecting placental dysfunction. No important association was found between third-trimester FIR and other outcomes including stillbirth, SGA and low Apgar score at 5 min, although the certainty of evidence for these associations was low [27].

A number of methodological differences likely account for the contrasting findings and interpretations. Some of these are related to the inclusion criteria, variations in the definitions of cases and calculation of a drop in IR, and variations in the definitions of composite markers of placental dysfunction. In particular, studies varied in the inclusion and the proportion of individuals with type 1, type 2, and gestational diabetes. Definitions of exposure (fall in IR) ranged from a decrease of ≥15% after 36 weeks [21], a decrease of ≥15% in weight-adjusted basal insulin after 30 weeks [18], a ≥15% decrease in total insulin dose from the peak dose in the third trimester to the lowest dose in the third trimester [19,20,25], ≥20% from maximal total insulin dose during pregnancy [26], and reduction of at least 30% of insulin intake during a 2 week period [24]. In terms of composite outcomes, components representing placental dysfunction varied between studies. One study included five components: preeclampsia, SGA, preterm birth (<30 weeks), placental abruption, and stillbirth (>20 weeks) [19], while another study included maternal hypertensive complications, intrauterine growth restriction, oligohydramnios or polyhydramnios, non-reassuring fetal heart tracings during routine antenatal testing, biophysical profile score less than 8/8, or abnormal umbilical artery dopplers (defined as S/D ratio > 95th percentile for gestational age or absent/reversed end-diastolic flow [23].

While most patients with pre-existing diabetes have increased IR in the third trimester [9,10,12,17], a small portion demonstrate a decrease in third-trimester IR [8,17,18,21]. The reason why some patients have a third-trimester decrease in IR is unclear, but it may be related to a longer duration of diabetes [9,21] and the assumption that patients with long-standing diabetes may have poorer placental function, and therefore produce lesser amounts of diabetogenic placental hormones [9]. However, in most instances, this decrease in IR was not associated with any adverse fetal or maternal outcomes [17,18,21].

The association between clinical manifestations of placental insufficiency and falling IR is complex, especially in terms of determining temporality. Clinical outcomes such as preeclampsia and fetal growth restriction are likely a result of a more chronic, pathological placental process that may begin before the third trimester [28,29]. Falling IR, therefore, could be part of the same placental process or a manifestation of preeclampsia and fetal growth restriction rather than the cause. Previous studies have not considered temporality while determining associations. To address this, we included in our primary composite outcome, only the acute manifestations of placental dysfunction or fetal distress that occurred after the fall in IR. Based on the results of our study, we were not able to demonstrate an association between a third-trimester drop in total or basal IR and the subsequent occurrence of adverse pregnancy outcomes, even when the IR dropped by as much as ≥30%. These findings are supported by a meta-analysis of the published literature on the topic that summarizes all available evidence [27].

Recent research also demonstrates that pathological placental processes or changes in pre-existing diabetes and gestational diabetes are highly heterogeneous, involving diverse changes in placental structure, angiogenesis, and inflammatory pathways. This includes trophoblastic and vascular remodeling in gestational diabetes that may influence placental hormone production and fetal outcomes [30] or significant modifications in placental vascular architecture and function in pre-existing diabetic pregnancies [31]. These findings also suggest that changes in IR alone may not reliably reflect the full spectrum of placental adaptations or dysfunction, particularly in a population with diverse diabetes types and durations.

Third trimester stillbirths are probably the most devastating complications in pregnancies complicated by diabetes, [32,33] and in our study we report three stillbirths that occurred between 36 + 0 and 38 + 5 weeks. However, all three of these occurred in pregnancies where there was no drop in IR. The fear of unexplained stillbirth is one of the main reasons why preterm induction and delivery are sometimes considered in cases of falling IR, and this contributes to the observed increase in preterm birth rates [34]. We acknowledge that clinical decisions in the light of falling third-trimester IR are complex and need a nuanced and individualized approach.

However, based on our study and a summary of available evidence, we cannot recommend routine delivery before term solely due to drop in IR, especially as babies delivered at late preterm and early term gestations can have negative long-term neurological impairments, developmental disabilities and behavioral problems [35,36,37,38,39], and stillbirths may also occur in pregnancies with no documented drop in IR. Instead, it would be prudent to recommend appropriate maternal and fetal surveillance based on the full clinical context, such as accompanying diagnoses of fetal growth concerns (growth restriction as well as macrosomia), and hypertensive disorders of pregnancy.

The limitations of our study include the retrospective study design and the rare nature of adverse outcomes which limit the statistical power to detect small or infrequent but clinically significant risks. While our model included key clinical variables, we recognize that other unmeasured confounders for example, gestational weight gain, insulin sensitivity changes, or use of adjunct therapies like metformin may contribute to the outcome and the omitted variable bias could be a potential limitation of our analysis. However, prior to the publication of the Metformin in women with type 2 diabetes in pregnancy (MiTy) trial in 2020 [40], we did not use metformin very often in pregnant people with type 2 diabetes. Participants were recruited for the MiTy trial between 25 May 2011 and 11 Oct 2018, so while this is a limitation, it likely does not affect the results significantly. We acknowledge that insulin resistance increases as pregnancy progresses, however, we have no way of measuring this in a usual clinical setting. We assume that, with falling IR, this reflects changes in insulin sensitivity assumed to be due to placental aging and a fall in hormone production, but a direct measurement of insulin resistance is not usually possible. We also acknowledge that the period of the study from 2000 to 2016 is a long study window during which temporal management changes could have been introduced (e.g., new insulin analogs, evolving standards of antenatal care). However, rapid-acting analogs (Humalog, Aspart, Glulisine) were introduced into Canada in the late 1990s while long-acting analogs (detemir and glargine) were introduced in the early 2000s. The short-acting insulins conferred better glucose levels by mimicking post-prandial glucose excursions better than previously, and the long-acting insulins conferred a flatter profile, thus leading to less hypoglycemia. While the treatment targets did not change over that time, these insulins allowed for better achievement of the pregnancy goals with less hypoglycemia. Since the study time period started around the same time as the insulins were available, it should not have posed a problem. Finally, while our study results did not show a statistically significant association and adverse pregnancy outcomes, we acknowledge that the rare nature of the study outcomes limits the statistical power to detect the difference in the composite outcome. Therefore, the possibility of type II errors should be considered when interpreting the results.

Despite these limitations, in our study, which is the largest study on the topic, we ensured that the composite outcome only included components that can be attributed to placental dysfunction, and which occurred following the drop in IR, thereby addressing the issue of temporality that has not been addressed adequately in published studies. To better understand the association between IR and adverse pregnancy outcomes, future research should involve larger prospective multicenter studies, separate analyses of patients with type 1 and type 2 diabetes, and a standardized approach to reporting and calculating changes in insulin requirements in order to reduce heterogeneity.

In conclusion, in a single-center retrospective cohort study involving 350 pregnancies with pre-existing diabetes, an isolated finding of a 15% or 30% drop in third trimester total or basal insulin requirements was not associated with adverse pregnancy outcomes. Given these findings and a summary of the available literature, the sole finding of a third-trimester drop in IR in the absence of other clinical findings should not be considered an indication for delivery, but instead, clinicians should recognize the clinical uncertainty presented by an otherwise-unexplained drop in IR, and consider an individualized care plan that includes appropriate maternal and fetal surveillance based on the clinical context and the long-term risks of late preterm and early term births.

## Figures and Tables

**Table 1:** Maternal characteristics with or without a ≥15% drop in total daily insulin requirements.

Variable	Drop ≥ 15% (Cases)	Drop ≤ 15% (Controls)	*p* Value
	*N* = 54	*N* = 296	
Age, mean (SD)	31.78 (6.0)	33.28 (5.4)	0.067
Type 1 diabetes, *n* (%)	36 (66.6)	110 (37.1)	**<0.001**
Type 2 diabetes, *n* (%)	18 (33.3)	186 (62.8)	**<0.001**
Nulliparous, *n* (%)	19 (35.2)	164 (55.4)	**0.010**
BMI, mean (SD)	26.69 (7.0)	29.73 (7.3)	**0.005**
Pre-pregnancy HbA1c, mean, (SD)	7.53 (1.7)	7.46 (1.8)	0.788
Microvascular disease, *n* (%)	8 (14.8)	48 (16.2)	
Nephropathy, *n* (%)	2 (3.7)	11 (3.7)	1.000
Retinopathy, *n* (%)	5 (9.3)	27 (9.1)	1.000
Neuropathy, *n* (%)	2 (3.7)	10 (3.4)	1.000
Pre-existing hypertension, *n* (%)	5 (9.3)	61 (20.6)	0.076
Smoking status, *n* (%)	8 (14.8)	41 (13.8)	1.000

BMI = body mass index, SD = standard deviation. Values in bold are statistically significant (*p* < 0.05)

**Table 2:** Pregnancy outcomes (with or without a ≥15% drop in total daily insulin requirements).

Variable	Drop ≥ 15% (Cases)	Drop ≤ 15% (Controls)	*p* Value
	*N* = 54	*N* = 296	
Composite Outcome	9 (16.6)	63 (21.2)	0.582
***Component outcomes***			
Stillbirth, *n* (%)	0 (0.0)	3 (1.10)	1.0
Spontaneous preterm birth or preterm premature rupture of membranes, *n* (%)	1 (1.5)	17 (6.0)	0.392
Iatrogenic preterm birth for fetal wellbeing concerns, *n* (%)	0 (0.0)	11 (3.7)	0.309
Emergency cesarean for fetal wellbeing, *n* (%)	8 (14.8)	38 (12.9)	0.867
***Secondary outcomes***			
Hypertensive disorders of pregnancy, *n* (%)	10 (18.5)	61 (20.6)	0.867
Gestational age at birth, mean (SD)	38.18 (1.16)	38.00 (1.48)	0.393
Birthweight, mean (SD)	3472.19 (704.79)	3382.19 (686.88)	0.379
Birthweight below 10th centile, *n* (%)	3 (5.6)	24 (8.1)	0.707
Neonatal intensive care unit admission, *n* (%)	15 (27.8)	65 (22.0)	0.455

SD = standard deviation.

**Table 3:** Maternal characteristics (with or without ≥30% drop in total daily insulin requirements).

Variable	Drop ≥ 30% (Cases)	Drop ≤ 30% (Controls)	*p* Value
	*N* = 22	*N* = 328	
Age, mean (SD)	31.32 (6.4)	33.16 (5.5)	0.131
Type 1 diabetes, *n* (%)	14 (63.6)	132 (40.4)	0.05
Type 2 diabetes, *n* (%)	8 (36.4)	196 (59.8)	0.05
Nulliparous, *n* (%)	8 (36.4)	175 (53.4)	0.185
BMI, mean (SD)	27.82 (6.9)	29.36 (7.4)	0.340
Pre-pregnancy HbA1c, mean, (SD)	7.58 (2.05)	7.46 (1.78)	0.776
Microvascular disease, *n* (%)			
Nephropathy, *n* (%)	1 (4.5)	12 (3.7)	1.000
Retinopathy, *n* (%)	1 (4.5)	31 (9.15)	0.696
Neuropathy, *n* (%)	1 (4.5)	11 (3.4)	1.000
Pre-existing hypertension, *n* (%)	4 (18.2)	62 (18.9)	1.000
Smoking status, *n* (%)	4 (18.1)	45 (13.7)	1.000

BMI = body mass index, SD = standard deviation.

**Table 4:** Pregnancy outcomes (with or without a ≥30% drop in total daily insulin requirements).

Variable	Drop ≥ 30% (Cases)	Drop ≤ 30% (Controls)	*p* Value
	*N* = 22	*N* = 328	
Composite Outcome	5 (22.7)	36 (10.9)	0.157
***Component outcomes***			
Stillbirth, *n* (%)	0 (0.0)	3 (0.9)	1.000
Spontaneous preterm birth or preterm premature rupture of membranes, *n* (%)	1 (4.5)	17 (5.2)	1.000
Iatrogenic preterm birth for fetal wellbeing concerns, *n* (%)	0 (0.0)	11 (3.4)	0.807
Emergency cesarean for fetal wellbeing, *n* (%)	2 (9.1)	44 (13.5)	0.795
***Secondary outcomes***			
Hypertensive disorders of pregnancy, *n* (%)	5 (22.7)	66 (20.1)	0.984
Gestational age at birth, mean (SD)	37.70 (1.33)	38.05 (1.44)	0.278
Birthweight, mean (SD)	3271.55 (565.00)	3404.50 (696.92)	0.382
Birthweight below 10th centile, *n* (%)	0 (5.6)	27 (8.3)	0.322
Neonatal intensive care unit admission, *n* (%)	7 (31.8)	73 (22.3)	0.445

SD = standard deviation.

## Data Availability

All supporting data are available within the article and its Supplementary Materials. Further inquiries can be directed to the corresponding authors.

## Supplementary Materials

The following supporting information can be downloaded at: https://www.mdpi.com/article/10.3390/jcm14217737/s1, Table S1: Maternal characteristics stratified using ≥15% thresholds of total daily Insulin Requirements with Type 1 diabetes; Table S2: Maternal characteristics stratified using ≥15% thresholds of total daily Insulin Requirements with Type 2 diabetes; Table S3: Pregnancy outcomes stratified using ≥15% thresholds of total daily Insulin Requirement with Type 1 diabetes; Table S4: Pregnancy outcomes stratified using ≥15% thresholds of total daily Insulin Requirement with Type 2 diabetes; Table S5: Maternal characteristics stratified using ≥30% thresholds of total daily Insulin with Type 1 diabetes; Table S6: Maternal characteristics stratified using ≥30% thresholds of total daily Insulin with Type 2 diabetes; Table S7: Pregnancy outcomes stratified using ≥30% thresholds of total daily Insulin with Type 1 diabetes; Table S8: Pregnancy outcomes stratified using ≥30% thresholds of total daily Insulin with Type 2 diabetes; Table S9: Maternal characteristics (with or without ≥15% drop in total basal insulin requirement); Table S10: Pregnancy outcomes (with or without ≥15% drop in total basal insulin requirement); Table S11: Maternal Characteristics for patients with and without a ≥30% drop in basal insulin requirements; Table S12: Pregnancy Outcomes in patients with and without a ≥30% drop in basal insulin requirements.

## References

[1] Albrecht S.S., Kuklina E.V., Bansil P., Jamieson D.J., Whiteman M.K., Kourtis A.P., Posner S.F., Callaghan W.M. (2010). Diabetes trends among delivery hospitalizations in the U.S., 1994–2004. Diabetes Care.

[2] Ngwezi D.P., Savu A., Yeung R.O., Butalia S., Kaul P. (2023). Temporal Trends in Type 1, Type 2, and Gestational Diabetes in Pregnancy: Impact of Rural Residence, Ethnicity, and Material Deprivation. Can. J. Diabetes.

[3] Gazis D., Tranidou A., Siargkas A., Apostolopoulou A., Koutsouki G., Goulis D.G., Tsakalidis C., Tsakiridis I., Dagklis T. (2025). Pregestational Diabetes Mellitus and Adverse Perinatal Outcomes: A Systematic Review and Meta-Analysis. J. Clin. Med..

[4] Gualdani E., Di Cianni G., Seghieri M., Francesconi P., Seghieri G. (2021). Pregnancy outcomes and maternal characteristics in women with pregestational and gestational diabetes: A retrospective study on 206,917 singleton live births. Acta Diabetol..

[5] American Diabetes Association (2020). 14. Management of Diabetes in Pregnancy: Standards of Medical Care in Diabetes-2020. Diabetes Care.

[6] (2018). American College of Obstetricians and Gynecologists’ Committee on Practice Bulletins—Obstetrics. ACOG Practice Bulletin No. 201: Pregestational Diabetes Mellitus. Obstet. Gynecol..

[7] American Diabetes Association Professional Practice Committe (2023). 15. Management of Diabetes in Pregnancy: Standards of Care in Diabetes—2024. Diabetes Care.

[8] Rayburn W., Piehl E., Lewis E., Schork A., Sereika S., Zabrensky K. (1985). Changes in insulin therapy during pregnancy. Am. J. Perinatol..

[9] Kambara M., Yanagisawa K., Tanaka S., Suzuki T., Babazono T. (2019). Changes in insulin requirements during pregnancy in Japanese women with type 1 diabetes. Diabetol. Int..

[10] Roeder H.A., Moore T.R., Ramos G.A. (2012). Insulin pump dosing across gestation in women with well-controlled type 1 diabetes mellitus. Am. J. Obstet. Gynecol..

[11] Langer O., Anyaegbunam A., Brustman L., Guidetti D., Levy J., Mazze R. (1988). Pregestational diabetes: Insulin requirements throughout pregnancy. Am. J. Obstet. Gynecol..

[12] Garcia-Patterson A., Gich I., Amini S.B., Catalano P.M., de Leiva A., Corcoy R. (2010). Insulin requirements throughout pregnancy in women with type 1 diabetes mellitus: Three changes of direction. Diabetologia.

[13] Ryan E.A., Enns L. (1988). Role of gestational hormones in the induction of insulin resistance. J. Clin. Endocrinol. Metab..

[14] Tyson J.E., Hwang P., Guyda H., Friesen H.G. (1972). Studies of prolactin secretion in human pregnancy. Am. J. Obstet. Gynecol..

[15] Burke C.W., Roulet F. (1970). Increased exposure of tissues to cortisol in late pregnancy. Br. Med. J..

[16] Cunningham F.G., Leveno K.J., Dashe J.S., Hoffman B.L., Spong C.Y., Casey B.M. (2022). Williams Obstetrics, 26e.

[17] Steel J.M., Johnstone F.D., Hume R., Mao J.H. (1994). Insulin requirements during pregnancy in women with type I diabetes. Obstet. Gynecol..

[18] Achong N., Callaway L., d’Emden M., McIntyre H.D., Lust K., Barrett H.L. (2012). Insulin requirements in late pregnancy in women with type 1 diabetes mellitus: A retrospective review. Diabetes Res. Clin. Pract..

[19] Padmanabhan S., Lee V.W., Mclean M., Athayde N., Lanzarone V., Khoshnow Q., Peek M.J., Cheung N.W. (2017). The Association of Falling Insulin Requirements with Maternal Biomarkers and Placental Dysfunction: A Prospective Study of Women with Preexisting Diabetes in Pregnancy. Diabetes Care.

[20] Padmanabhan S., McLean M., Cheung N.W. (2014). Falling insulin requirements are associated with adverse obstetric outcomes in women with preexisting diabetes. Diabetes Care.

[21] McManus R.M., Ryan E.A. (1992). Insulin requirements in insulin-dependent and insulin-requiring GDM women during final month of pregnancy. Diabetes Care.

[22] Plows J.F., Stanley J.L., Baker P.N., Reynolds C.M., Vickers M.H. (2018). The Pathophysiology of Gestational Diabetes Mellitus. Int. J. Mol. Sci..

[23] Wilkinson B., McDonnell M., Palermo N., Lassey S., Little S. (2021). Falling insulin requirement in late pregnancy: Association with obstetric and neonatal outcomes. J. Perinatol..

[24] Ram M., Feinmesser L., Shinar S., Maslovitz S. (2017). The importance of declining insulin requirements during pregnancy in patients with pre-gestational gestational diabetes mellitus. Eur. J. Obstet. Gynecol. Reprod. Biol..

[25] Padmanabhan S., Lee V., Mclean M., Athayde N., Lanzarone V., Peek M.J., Quinton A., Cheung N.W. (2022). The relationship between falling insulin requirements and serial ultrasound measurements in women with preexisting diabetes: A prospective cohort study. J. Matern.-Fetal Neonatal Med..

[26] Søholm J.C., Do N.C., Vestgaard M., Ásbjörnsdóttir B., Nørgaard S.K., Pedersen B.W., Storgaard L., Nielsen B.B., Holmager P., Ringholm L. (2022). Falling Insulin Requirement in Pregnant Women with Diabetes Delivering Preterm: Prevalence, Predictors, and Consequences. J. Clin. Endocrinol. Metab..

[27] D’Souza R., Ashraf R., Sayfi S., Prior A., Pihelgas P., Sanni O., Sayyar P., Alavifard S., Ospina M., Jain V. (2025). Falling third-trimester insulin requirements in diabetic pregnancies and adverse pregnancy outcomes: A systematic review and meta-analysis. J. Clin. Med..

[28] Vachon-Marceau C., Demers S., Markey S., Okun N., Girard M., Kingdom J., Bujold E. (2017). First-trimester placental thickness and the risk of preeclampsia or SGA. Placenta.

[29] Syngelaki A., Magee L.A., von Dadelszen P., Akolekar R., Wright A., Wright D., Nicolaides K.H. (2022). Competing-risks model for pre-eclampsia and adverse pregnancy outcomes. Ultrasound Obstet. Gynecol..

[30] Molitierno R., Imparato A., Iavazzo N., Salzillo C., Marzullo A., Laganà A.S., Etrusco A., Agrifoglio V., D’amato A., Renata E. (2025). Microscopic changes and gross morphology of placenta in women affected by gestational diabetes mellitus in dietary treatment: A systematic review. Open Med. (Wars).

[31] Huynh J., Dawson D., Roberts D., Bentley-Lewis R. (2015). A systematic review of placental pathology in maternal diabetes mellitus. Placenta.

[32] Mackin S.T., Nelson S.M., Wild S.H., Colhoun H.M., Wood R., Lindsay R.S. (2019). Factors associated with stillbirth in women with diabetes. Diabetologia.

[33] Page J.M., Allshouse A.A., Cassimatis I., Smid M.C., Arslan E., Thorsten V., Parker C., Varner M.W., Dudley D.J., Saade G.R. (2020). Characteristics of Stillbirths Associated with Diabetes in a Diverse U.S. Cohort. Obstet. Gynecol..

[34] Søholm J.C., Vestgaard M., Ásbjörnsdóttir B., Do N.C., Pedersen B.W., Storgaard L., Nielsen B.B., Ringholm L., Damm P., Mathiesen E.R. (2021). Potentially modifiable risk factors of preterm delivery in women with type 1 and type 2 diabetes. Diabetologia.

[35] Vohr B. (2013). Long-term outcomes of moderately preterm, late preterm, and early term infants. Clin. Perinatol..

[36] Dong Y., Chen S.J., Yu J.L. (2012). A systematic review and meta-analysis of long-term development of early term infants. Neonatology.

[37] Hirata K., Ueda K., Wada K., Ikehara S., Tanigawa K., Kimura T., Ozono K., Sobue T., Iso H. (2024). Neurodevelopmental outcomes at age 3 years after moderate preterm, late preterm and early term birth: The Japan Environment and Children’s Study. Arch. Dis. Child.-Fetal Neonatal Ed..

[38] Mitha A., Chen R., Razaz N., Johansson S., Stephansson O., Altman M., Bolk J. (2024). Neurological development in children born moderately or late preterm: National cohort study. BMJ.

[39] Chan E., Leong P., Malouf R., Quigley M.A. (2016). Long-term cognitive and school outcomes of late-preterm and early-term births: A systematic review. Child Care Health Dev..

[40] Feig D.S., Donovan L.E., Zinman B., Sanchez J.J., Asztalos E., Ryan E.A., Fantus I.G., Hutton E., Armson A.B., Lipscombe L.L. (2020). Metformin in women with type 2 diabetes in pregnancy (MiTy): A multicentre, international, randomised, placebo-controlled trial. Lancet Diabetes Endocrinol..

