# Supplementary material for: Falling Third Trimester Insulin Requirements and Adverse Pregnancy Outcomes in Individuals with Pre-Existing Diabetes: A Retrospective Cohort Study
Source: J Clin Med. 2025 Oct 31;14(21):7737. doi: 10.3390/jcm14217737 (PMC12610794; doi:10.3390/jcm14217737)
Supplement: Supplementary file 1 [file jcm-14-07737-s001.zip › Supplementary File S1.pdf]

Table S1. Maternal characteristics stratified using ≥15% thresholds of total daily Insulin Requirements with Type 1 diabetes

Variable	Drop ≥15% (Cases)	Drop ≤15% (Controls)	P Value
	N=36	N=110	
Age, mean (SD)	30.14 (6.4)	30.69 (5.2)	0.605
Nulliparous, n (%)	9 (25.0)	54 (49.1)	**0.019**
BMI, mean (SD)	24.26 (4.1)	26.92 (6.7)	**0.029**
Pre-pregnancy HbA1c, mean, (SD)	7.54 (1.7)	7.46 (1.8)	0.810
Microvascular disease, n (%)			
Nephropathy, n (%)	0 (0.0)	9 (8.2)	0.170
Retinopathy, n (%)	5 (13.9)	19 (17.3)	0.829
Neuropathy, n (%)	1 (2.8)	6 (5.5)	0.839
Pre-existing hypertension, n (%)	1 (2.8)	13 (11.8)	0.203
Smoking status, n (%)	6 (16.6)	9 (8.1)	0.759

BMI = body mass index, SD = standard deviation.

Table S2. Maternal characteristics stratified using ≥15% thresholds of total daily Insulin Requirements with Type 2 diabetes

Variable	Drop ≥15% (Cases)	Drop ≤15% (Controls)	P Value
	N=18	N=186	
Age, mean (SD)	35.06 (3.30)	34.81 (4.95)	0.835
Nulliparous, n (%)	10 (55.6)	110 (59.1)	0.965
BMI, mean (SD)	31.71 (9.01)	31.32 (7.17)	0.836
Pre-pregnancy HbA1c, mean, (SD)	7.51 (1.8)	7.46 (1.8)	0.912
Microvascular disease, n (%)	3 (16.6)	14 (7.5)	
Nephropathy, n (%)	2 (11.1)	2 (1.1)	**0.041**
Retinopathy, n (%)	0 (0.0)	8 (4.3)	0.793
Neuropathy, n (%)	1 (5.6)	4 (2.2)	0.925
Pre-existing hypertension, n (%)	4 (22.2)	48 (25.8)	0.960
Smoking status, n (%)	2 (11.1)	32 (20.7)	1.000

BMI = body mass index, SD = standard deviation.
