# Supplementary material for: Falling Third Trimester Insulin Requirements and Adverse Pregnancy Outcomes in Individuals with Pre-Existing Diabetes: A Retrospective Cohort Study
Source: J Clin Med. 2025 Oct 31;14(21):7737. doi: 10.3390/jcm14217737 (PMC12610794; doi:10.3390/jcm14217737)
Supplement: Supplementary file 1 [file jcm-14-07737-s001.zip › Supplementary File S2.pdf]

Table S3. Pregnancy outcomes stratified using ≥15% thresholds of total daily Insulin Requirement with Type 1 diabetes

Variable	Drop ≥15% (Cases) N=36	Drop ≤15% (Controls) N=110	P Value
Composite Outcome	7 (19.4)	20 (18.2)	1.000
Component outcomes			
Stillbirth, n (%)	0 (0.0)	0 (0)	NA
Spontaneous preterm birth or preterm premature rupture of membranes, n (%)	1 (2.8)	10 (9.1)	0.339
Iatrogenic preterm birth for fetal wellbeing concerns, n (%)	0 (0.0)	2 (1.8)	1.000
Emergency caesarean for fetal wellbeing, n (%)	6 (16.7)	10 (9.1)	0.339
Secondary outcomes			
Hypertensive disorders of pregnancy, n (%)	7 (19.4)	26 (23.6)	0.770
Gestational age at birth, mean (SD)	38.00 (1.20)	37.66 (1.64)	0.252
Birthweight, mean (SD)	3671.56 (734.67)	3556.49 (731.88)	0.415
Birthweight below 10th centile, n (%)	1 (2.8)	4 (3.6)	1.000
Neonatal intensive care unit admission, n (%)	12 (33.3)	33 (30.0)	0.867

SD = standard deviation.

Table S4. Pregnancy outcomes stratified using ≥15% thresholds of total daily Insulin Requirement with Type 2 diabetes

Variable	Drop ≥15% (Cases)	Drop ≤15% (Controls)	P Value
	N=18	N=186	
Composite Outcome	2 (11.1)	43 (23.1)	0.381
Component outcomes			
Stillbirth, n (%)	0 (0.0)	3 (1.6)	1.000
Spontaneous preterm birth or preterm premature rupture of membranes, n (%)	0 (0.0)	7 (3.8)	0.873
Iatrogenic preterm birth for fetal wellbeing concerns, n (%)	0 (0.0)	9 (4.9)	0.721
Emergency caesarean for fetal wellbeing, n (%)	2 (11.1)	28 (15.1)	0.911
Secondary outcomes			
Hypertensive disorders of pregnancy, n (%)	3 (16.7)	35 (18.8)	1.000
Gestational age at birth, mean (SD)	38.53 (1.01)	38.20 (1.34)	0.303
Birthweight, mean (SD)	3073.44 (429.97)	3278.56 (638.42)	0.184
Birthweight below 10th centile, n (%)	2 (11.1)	20 (10.8)	1.000
Neonatal intensive care unit admission, n (%)	3 (16.7)	32 (17.3)	1.000

SD = standard deviation.
