# Supplementary material for: Falling Third Trimester Insulin Requirements and Adverse Pregnancy Outcomes in Individuals with Pre-Existing Diabetes: A Retrospective Cohort Study
Source: J Clin Med. 2025 Oct 31;14(21):7737. doi: 10.3390/jcm14217737 (PMC12610794; doi:10.3390/jcm14217737)
Supplement: Supplementary file 1 [file jcm-14-07737-s001.zip › Supplementary File S3.pdf]

Table S5. Maternal characteristics stratified using ≥30% thresholds of total daily Insulin with Type 1 diabetes

Variable	Drop ≥30% (Cases)	Drop ≤30% (Controls)	P Value
	N=14	N=132	
Age, mean (SD)	28.36 (5.9)	30.79 (5.5)	0.118
Nulliparous, n (%)	2 (14.3)	61 (46.2)	**0.044**
BMI, mean (SD)	24.64 (4.36)	26.42 (6.43)	0.315
Pre-pregnancy HbA1c, mean, (SD)	7.92 (2.38)	7.43 (1.68)	0.346
Microvascular disease, n (%)			
Nephropathy, n (%)	0 (0.0)	9 (6.8)	0.671
Retinopathy, n (%)	1 (7.1)	23 (17.4)	0.543
Neuropathy, n (%)	0 (0.0)	7 (5.3)	0.822
Pre-existing hypertension, n (%)	0 (0.0)	14 (10.6)	0.421
Smoking status, n (%)	3 (21.4)	12 (9.1)	1.000

BMI = body mass index, SD = standard deviation.

Table S6. Maternal characteristics stratified using ≥30% thresholds of total daily Insulin with Type 2 diabetes

Variable	Drop ≥30% (Cases)	Drop ≤30% (Controls)	P Value
	N=8	N=196	
Age, mean (SD)	36.50 (3.38)	34.76 (4.86)	0.318
Nulliparous, n (%)	6 (75.0)	114 (58.2)	0.561
BMI, mean (SD)	33.38 (7.21)	31.27 (7.33)	0.426
Pre-pregnancy HbA1c, mean, (SD)	6.96 (1.14)	7.48 (1.87)	0.461
Microvascular disease, n (%)			
Nephropathy, n (%)	1 (12.5)	3 (1.5)	0.372
Retinopathy, n (%)	0 (0.0)	8 (4.1)	1.000
Neuropathy, n (%)	1 (12.5)	4 (2.0)	0.478
Pre-existing hypertension, n (%)	4 (50)	48 (24.5)	0.227
Smoking status, n (%)	1 (12.5)	33 (16.8)	1.000

BMI = body mass index, SD = standard deviation.
