# Supplementary material for: Falling Third Trimester Insulin Requirements and Adverse Pregnancy Outcomes in Individuals with Pre-Existing Diabetes: A Retrospective Cohort Study
Source: J Clin Med. 2025 Oct 31;14(21):7737. doi: 10.3390/jcm14217737 (PMC12610794; doi:10.3390/jcm14217737)
Supplement: Supplementary file 1 [file jcm-14-07737-s001.zip › Supplementary File S4.pdf]

Table S7. Pregnancy outcomes stratified using ≥30% thresholds of total daily Insulin with Type 1 diabetes

Variable	Drop ≥30% (Cases) N=14	Drop ≤30% (Controls) N=132	P Value
Composite Outcome	3 (21.4)	24 (18.2)	1.000
Component outcomes			
Stillbirth, n (%)	0 (0.0)	0 (0.0)	NA
Spontaneous preterm birth or preterm premature rupture of membranes, n (%)	1 (7.1)	3 (7.6)	1.000
Iatrogenic preterm birth for fetal wellbeing concerns, n (%)	0 (0.0)	2 (1.5)	1.000
Emergency caesarean for fetal wellbeing, n (%)	2 (14.3)	14 (10.6)	1.000
Secondary outcomes			
Hypertensive disorders of pregnancy, n (%)	5 (35.7)	28 (21.2)	0.369
Gestational age at birth, mean (SD)	37.52 (1.59)	37.77 (1.55)	0.572
Birthweight, mean (SD)	3405.50 (636.93)	3603.89 (740.66)	0.336
Birthweight below 10th centile, n (%)	0 (0.0)	5 (3.8)	1.000
Neonatal intensive care unit admission, n (%)	5 (35.7)	42 (31.8)	0.910

SD = standard deviation.

Table S8. Pregnancy outcomes stratified using ≥30% thresholds of total daily Insulin with Type 2 diabetes

Variable	Drop ≥30% (Cases) N=8	Drop ≤30% (Controls) N=196	P Value
Composite Outcome	0 (0.0)	12 (23)	0.271
Component outcomes			
Stillbirth, n (%)	0 (0.0)	3 (1.5)	1.000
Spontaneous preterm birth or preterm premature rupture of membranes, n (%)	0 (0.0)	7 (3.6)	1.000
Iatrogenic preterm birth for fetal wellbeing concerns, n (%)	0 (0.0)	9 (4.6)	1.000
Emergency caesarean for fetal wellbeing, n (%)	0 (0.0)	30 (15.4)	1.000
Secondary outcomes			

Hypertensive disorders of pregnancy, n (%)	0 (0.0)	38 (19.4)	0.359
Gestational age at birth, mean (SD)	38.02 (0.63)	38.24 (1.34)	0.657
Birthweight, mean (SD)	3037.12 (324.95)	3269.53 (632.75))	0.304
Birthweight below 10th centile, n (%)	0 (0.0)	22 (11.3)	0.670
Neonatal intensive care unit admission, n (%)	2 (25.0)	33 (16.9)	0.908

SD = standard deviation.
