# Supplementary material for: Falling Third Trimester Insulin Requirements and Adverse Pregnancy Outcomes in Individuals with Pre-Existing Diabetes: A Retrospective Cohort Study
Source: J Clin Med. 2025 Oct 31;14(21):7737. doi: 10.3390/jcm14217737 (PMC12610794; doi:10.3390/jcm14217737)
Supplement: Supplementary file 1 [file jcm-14-07737-s001.zip › Supplementary File S5.pdf]

Table S9. Maternal characteristics (with or without ≥ 15% drop in total basal insulin requirement)

Variable	Drop ≥ 15% (Cases)	Drop ≤15% (Controls)	P Value
	N=50	N=300	
Age, mean (SD)	32.22 (6.2)	33.18 (5.4)	0.255
Type 2 diabetes, n (%)	24 (48.0)	180 (60.0)	0.150
Nulliparous, n (%)	25 (50.0)	158 (52.70)	0.844
BMI, mean (SD)	28.63 (7.9)	29.36 (7.2)	0.530
Pre-pregnancy HbA1c, mean, (SD)	7.59 (1.6)	7.45 (1.80)	0.662
Microvascular disease, n (%)	2 (4)	55 (18.3)	
• Nephropathy, n (%)	0 (0)	13 (4.30)	0.273
• Retinopathy, n (%)	1 (2.0)	31 (10.3)	0.104
• Neuropathy, n (%)	1 (2.0)	11 (3.70)	0.857
Pre-existing hypertension, n (%)	4 (8.0)	62 (20.7)	0.054
Smoking status	9 (18)	40 (13.3)	0.318

BMI = body mass index, SD = standard deviation.

Table S10. Pregnancy outcomes (with or without ≥ 15% drop in total basal insulin requirement)

Variable	Drop ≥ 15% (Cases)	Drop ≤15% (Controls)	P Value
**Composite Outcome**	**9**	**63**	**0.63**
**Component outcomes**			
1. Stillbirth, n (%)	1 (2.0)	2 (0.7)	0.895
2. Spontaneous preterm birth or preterm premature rupture of membranes, n (%)	2 (4.0)	16 (5.3)	0.961
3. Iatrogenic preterm birth for fetal wellbeing concerns, n (%)	0 (0.0)	11 (3.7)	0.357
4. Emergency caesarean for fetal wellbeing, n (%)	6 (12.2)	40 (13.3)	1.000
**Secondary outcomes**			
Hypertensive disorders of pregnancy, n (%)	8 (16.0)	63 (21.0)	0.533
Gestational age at birth, mean (SD)	38.18 (1.06)	38.00 (1.49)	0.433
Birthweight, mean (SD)	3396.51 (544.14)	3396.05 (711.10)	0.997
Birthweight below 10th centile, n (%)	1 (2.0)	26 (8.7)	0.186
Neonatal intensive care unit admission, n (%)	11 (22.4)	69 (23.0)	1.000

SD = standard deviation.
