# Supplementary material for: Falling Third Trimester Insulin Requirements and Adverse Pregnancy Outcomes in Individuals with Pre-Existing Diabetes: A Retrospective Cohort Study
Source: J Clin Med. 2025 Oct 31;14(21):7737. doi: 10.3390/jcm14217737 (PMC12610794; doi:10.3390/jcm14217737)
Supplement: Supplementary file 1 [file jcm-14-07737-s001.zip › Supplementary File S6.pdf]

Table S11. Maternal Characteristics for patients with and without a ≥30% drop in basal insulin requirements.

Variable	Drop ≥ 30% (Cases)	Drop ≤ 30% (Control)	P Value
	N=17	N=333	
Age, mean (SD)	31.82 (5.50)	33.11 (5.5)	0.352
Nulliparous, n (%)	9 (52.9)	174 (52.3)	1
BMI, mean (SD)	29.76 (7.45)	29.24 (7.34)	0.773
Pre-pregnancy HbA1c, mean, (SD)	6.69 (0.57)	7.51 (1.83)	0.095
Microvascular disease, n (%)	1 (5.9)	56 (16.8)	
• Nephropathy, n (%)	0 (0.0)	13 (3.9)	0.863
• Retinopathy, n (%)	0 (0.0)	32 (9.6)	0.363
• Neuropathy, n (%)	1 (5.9)	11 (3.3)	1
Pre-existing hypertension, n (%)	2 (11.8)	64 (19.2)	0.654
Smoking status	3 (17.6)	46 (13.8)	1

SD = standard deviation.

Table S12. Pregnancy Outcomes in patients with and without a ≥30% drop in basal insulin requirements.

Variable	Drop ≥ 30% (Cases)	Drop ≤ 30% (Control)	P Value
	N=17	N=333	
Composite Outcome	2	70	0.54
**Component Outcomes**			
• Stillbirth, n (%)	0 (0.0)	3 (0.9)	1
• Spontaneous pre-term birth or preterm premature rupture of membranes, n (%)	1 (5.9)	17 (5.1)	1
• Iatrogenic pre-term birth for fetal wellbeing, n (%)	0 (0.0)	11 (3.3)	0.959
• Emergency C-section for fetal wellbeing, n (%)	1 (5.9)	45 (13.6)	0.586
Hypertensive disorders of pregnancy, n (%)	2 (11.8)	69 (20.7)	0.558
GA at delivery, mean (SD)	37.84 (1.34)	38.04 (1.44)	0.587
Birthweight, mean (SD)	3327.00 (649.41)	3399.66 (692.15)	0.672
Birthweight below 10th centile, n (%)	0 (0.0)	27 (8.1)	0.448
NICU admission, n (%)	5 (29.4)	75 (22.6)	0.721
